# Cryptic extended brood care in the facultatively eusocial sweat bee *Megalopta genalis*

**DOI:** 10.1007/s00040-015-0409-3

**Published:** 2015-04-26

**Authors:** A. E. Quiñones, W. T. Wcislo

**Affiliations:** Theoretical biology group, University of Groningen, Nijenborgh 7, 9747 AG Groningen, The Netherlands; Smithsonian Tropical Research Institute, 0843-03092 Balboa, Apartado Republic of Panama

**Keywords:** Extended maternal care, Hygienic behavior, *Megalopta genalis*, Subsociality, Undertaking behavior, Eusociality

## Abstract

As a result of different brood cell provisioning strategies, nest-making insects may differ in the extent to which adults regularly provide extended parental care to their brood beyond nest defense. Mass-provisioning species cache the entire food supply needed for larval development prior to the oviposition and typically seal the brood cell. It is usually assumed that there is no regular contact between the adult(s) and brood. Here, we show that the bee, *Megalopta genalis*, expresses a form of cryptic brood care, which would not be observed during normal development. Following experimental injections of different provisioning materials into brood cells, foundresses reopened manipulated cells and the brood were aborted in some cases, implying that the foundresses assessed conditions within the cells. In aborted cells, foundresses sometimes laid a second egg after first removing dead larvae, previously stored pollen and contaminants. Our results show that hygienic brood care can be cryptic and hence may be more widespread than previously believed, lending support to the hypothesis that extended parental care is a preadaptation toward eusociality.

**Electronic supplementary material:**

The online version of this article (doi:10.1007/s00040-015-0409-3) contains supplementary material, which is available to authorized users.

## Introduction

Sociality has evolved in different clades of nest-making Hymenoptera, and their solitary ancestors likely differed in the extent to which adults provided extended parental care, apart from providing and protecting a nest (see Wheeler 1922; Wilson 1971; Michener 1974; West-Eberhard 1975; Alexander et al. 1991). “Mass-provisioning” species cache all food necessary for larval development prior to the oviposition (Michener 1974) and are generally thought to not provide additional care for individual brood, apart from defending the nest (Lin and Michener 1972). In contrast, in species with “progressive provisioning,” adults feed the larvae periodically throughout development. In such species, helping behavior may be advantageous, because in the event of the death of the egg-layer (or primary caregiver), related helpers assure that any initial investment in reproduction still pays off in terms of fitness (Queller 1989; 1994; Gadagkar 1990). In contrast, such insurance benefits (“assured fitness returns”) were thought to be of minimal importance in groups that are characterized by mass provisioning (but see Smith et al. 2003).

Halictine bees are mass provisioners (e.g., Michener 1974, 1990). After oviposition, the brood cells are usually closed off, isolating the brood from the adults, which has been assumed to limit adult–larval interactions. However, evidence has been accumulating that questions the assumption that social halictine bees lack extended post-oviposition parental care for individual brood. Plateaux-Quénu (2008) reviewed data showing that 17 species of eusocial halictines either leave brood cells open or reopen them. She hypothesized that active parental brood care (beyond nest defense) is a preadaptation to eusociality (also Alexander et al. 1991).

Here, we report modified brood care behavior associated with experimental injections of foreign material (supplemental food provisions) into brood cells of the facultatively eusocial sweat bee, *Megalopta genalis*. These manipulations triggered a form of maternal care that is not evident in the usual development of healthy brood cells, which has consequences for our understanding of the role of extended brood care in the evolution of insect sociality.

## Methods

### Study site and species

Collections and experiments were conducted on Barro Colorado Island (BCI; 9°09.754′N, 79°50.470′W), Panama. BCI is a lowland, semi-deciduous, moist forest with a pronounced dry season (Leigh 1999). Solitary and eusocial nests of *Megalopta genalis* co-occur within a single population (Wcislo et al. 2004; Smith et al. 2007, 2009; Kapheim et al. in press). *M. genalis* form nests in dead sticks in the forest understory. A tunnel with one entrance is excavated in a stick by a foundress. Females construct brood cells from chewed wood particles, which are then lined with hydrophobic secretions and provisioned with pollen and nectar. Once provisioning is completed, an egg is laid on the pollen ball and the brood cell is closed with a plug made of wood particles (Wcislo et al. 2004). Solitary nests result when female offspring disperse to mate, establish a nest, develop ovaries and become reproductively active (Kapheim et al. 2015 and references therein). In these nests, the foundresses carry out all tasks necessary for reproduction. In social nests, at least one female offspring does not disperse or develop ovaries, but takes over tasks related to nest maintenance and foraging (Smith et al. 2008); foundresses typically monopolize reproduction in social nests (Kapheim et al. 2013).

### Collection of natural nests

Nest collections were made during the dry season (January–March 2011) when the nests are relatively abundant and have many developing brood. Nest locations were recorded, and then the entrances were sealed with a cotton ball and transferred to a laboratory. In the laboratory, nests were opened and developing brood were kept in the laboratory until the pupal stage, when they were used to establish observation nests.

### Establishing observation nests

We set up 38 observation nests that were at least 10 m apart, and 23 nests produced brood. However, only 16 nests survived until the end of the study. The nests were not started at the same time, as setting them up depended on the availability of female pupae reared in the laboratory.

**Fig. 1:**
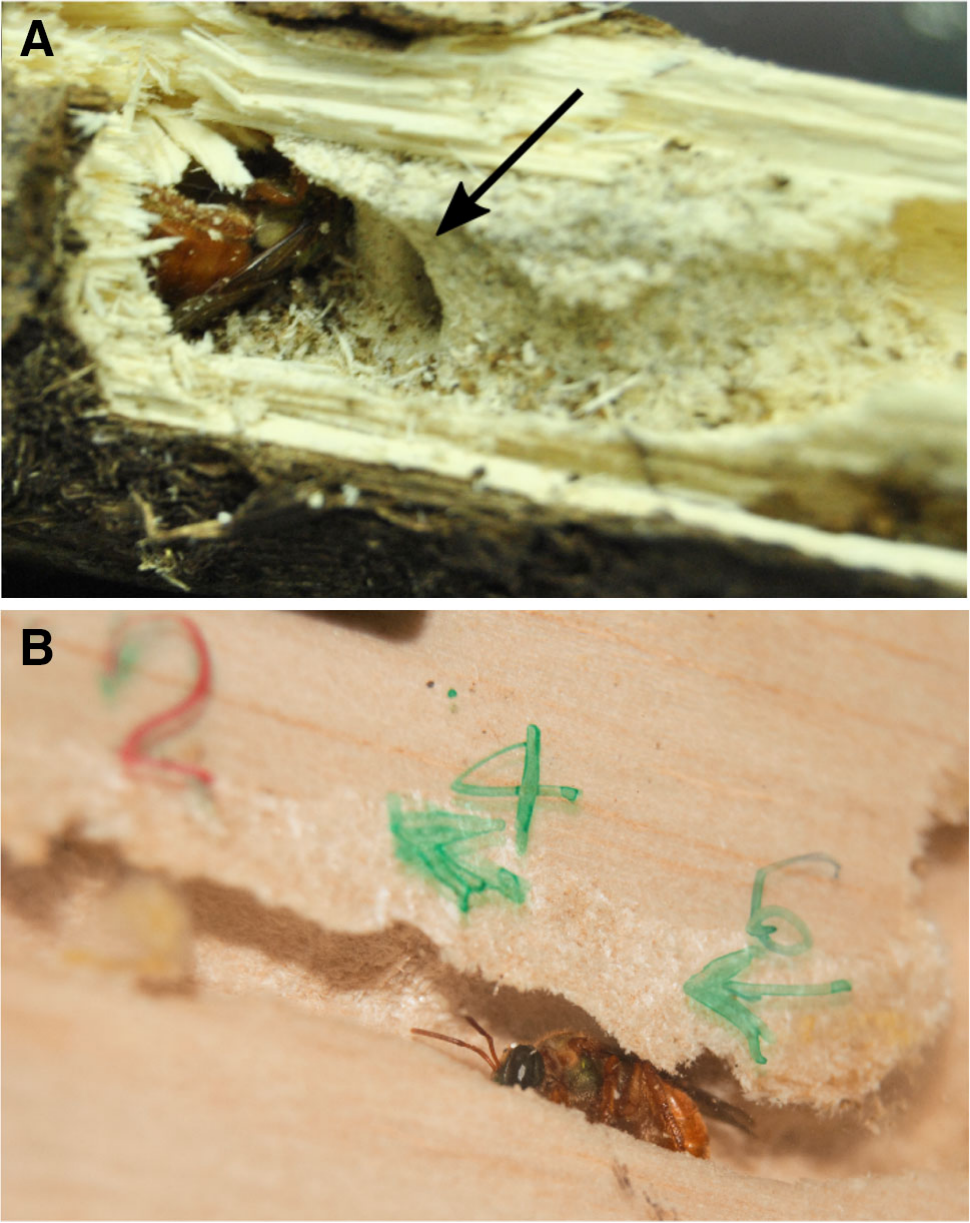
*Megalopta genalis* nests in dead sticks in the forest understory. The nest structure consists of a central tunnel dug by the foundress and adjacent brood cells. **a** A natural nest collected from the field. The* black arrow* points to an open brood cell. **b** An artificial observation nest with the foundress and three closed brood cells indicated by the numbers* 2*,* 4* and* 6*. The acrylic cover allowed us to mark and track the changes in each brood cell

Artificial observation nests were constructed following the procedure described in Wcislo and Gonzalez (2006), with subsequent modifications (Fig. 1; also see photo in supplementary materials of Kapheim et al. 2012). The nests consisted of a piece of balsa wood (~15 × 20 cm) with a central tunnel. The wood was covered with a piece of transparent acrylic and black cloth. Both the cloth and the acrylic could be removed to perform observations or to mark the newly emerged bees. These three components were covered with a plastic roof to prevent rain from getting inside and held together with binder clips. One female bee in the late pupal stage was placed in each observation nest, and the nest was subsequently hung on a wire from a vine in the forest at a height of approximately 1.5 m. We covered the top section of the wire with sticky Tanglefoot^®^ to prevent ant predation. Bees flew freely in the forest. Observation nests were checked every second day for collar ring construction, excavations inside the nest, presence or absence of the female and building of new brood cells. A plastic cup was installed just below the nest entrance, which collected all the material thrown out of the nest by the resident bee(s).

### Experimental manipulations

We did not set out initially to study cryptic brood care. Rather, our experimental manipulations represented an unsuccessful effort to assess the role of nutrition in caste determination. These manipulations consisted of the addition of one of the following to a provision mass: 40 µL of protein mix (9.8 % w/w), 40 µL of concentrated sucrose (200 % w/w) or 40 µL of distilled water as a control. The protein added was of a commercial brand of soy powder (“Trader Darwin’s Soy Protein Powder”); similar products have been successfully used to feed honey bees (Sereia et al. 2010). Provision masses were injected with a solution using a 1-ml syringe. In order to reduce contact with the egg or developing larvae, the injection was done in the last quarter of the brood cell, which is furthest away from the brood. The treatments were applied in random order to avoid bias from temporal factors. Experimental nests varied in the number of brood cells produced and the speed at which these were constructed. Therefore, the number of replicates varied among nests. Experiments were performed in recently founded nests; therefore, it was not possible to determine for all nests whether they would have developed into a social nest or remained solitary. Only three of the nests were clearly social, as females eclosed, stayed and help during the experimental phase. For each manipulative treatment, we injected 11 brood cells and a control group of 15 of brood cells was left without any injection (*N* = 48, total).

### Data analyses

Data analyses were conducted in R v. 3.1.2 (R Development Core Team 2008). We used generalized mixed effect models (GLMM) to analyze the effect of the treatments on the behavior of the foundresses toward the brood cell and on the final outcome of those cells. For all models, random effects were the nest ID (equivalent to foundress ID as there was one foundress per nest). The fixed effects used were the treatments described in the experimental manipulations. An extra fixed effect was included by grouping all the treatments that involved a manipulation (water control, addition of sucrose or protein) into a “manipulated” treatment, and contrasting this with brood cells that were un-manipulated; this grouping increased statistical power to distinguish whether any effect was caused by the manipulation or by the specific treatment.

We used three binomial response variables: (1) a cell was reopened or not, which is a measure of the effect of the treatments on modifying the foundress’ brood care behavior; (2) an adult bee emerged from the cell or not, regardless of development time, which ignores possible brood abortion and cell reuse; and (3) whether an adult emerged <40 days after the initial cell closure or not, which takes into account normal developmental time (~35 days) and accounts for possible cell reuse. As a continuous variable, we used the number of days between first closure of the brood cell and adult emergence. The three binomial variables were analyzed with a binomial distribution in the errors, while for developmental time, we used a Poisson distribution. Models were fitted using the “lme4” R package. We tested the significance of the fixed effects using the Likelihood ratio test between one model containing the effect and one without it. Once we found the minimal adequate model, we ran a Wald ${\chi^2}$ test to get the reported *p* values. *Posthoc* tests were performed using the glht function of the “multcomp” R package, which assesses the significance of the pairwise difference between the coefficients of the treatments. Some of the models suffered from complete separation (Heinze and Schemper 2002), which occurs when one of the levels of a fixed effect fully explains the binomial response variable. This prevents the algorithm from properly fitting the coefficient for this level. To overcome this problem, we fitted these models using the bglmer function of the “blme” R package. This function uses a similar algorithm to the “lmer4”, but allows the addition of a weak prior to the estimates of the coefficients, facilitating the estimation.

The data collected from the waste collection recipient were also analyzed using GLMM. These data represented a time series in which each data point corresponded to the presence or absence of a particular type of waste material discarded from the nest at each time point. The types of waste we looked for were pieces of the provisioned pollen mass and feces of the prepupal larvae. The random effect in these models was the nest from which the waste was collected. The fixed effects were the numbers of brood cells in different states inside the nest that the waste came from. The states used were open cell, closed cell, reopened cell and emerged adult. This way we assessed from which type of cell the waste was most likely coming from. Models were fitted using a binomial distribution. As the data correspond to time series, we accounted for autocorrelations by using the value of the response variable in the previous time step of the time series as one of the predictor variables. This estimation corresponds to fitting a Markov chain model where the coefficients determine the probability that the response variable changes from one state to the other in one time step. This was done first using GLMM in the “lmer4” package and confirmed fitting the same model using a Bayesian estimation of the parameters with the package “rjags”, which uses R as a platform to sample the posterior distributions using JAGS (Plummer 2003). The results of both models agreed qualitatively.

## Results

**Fig. 2:**
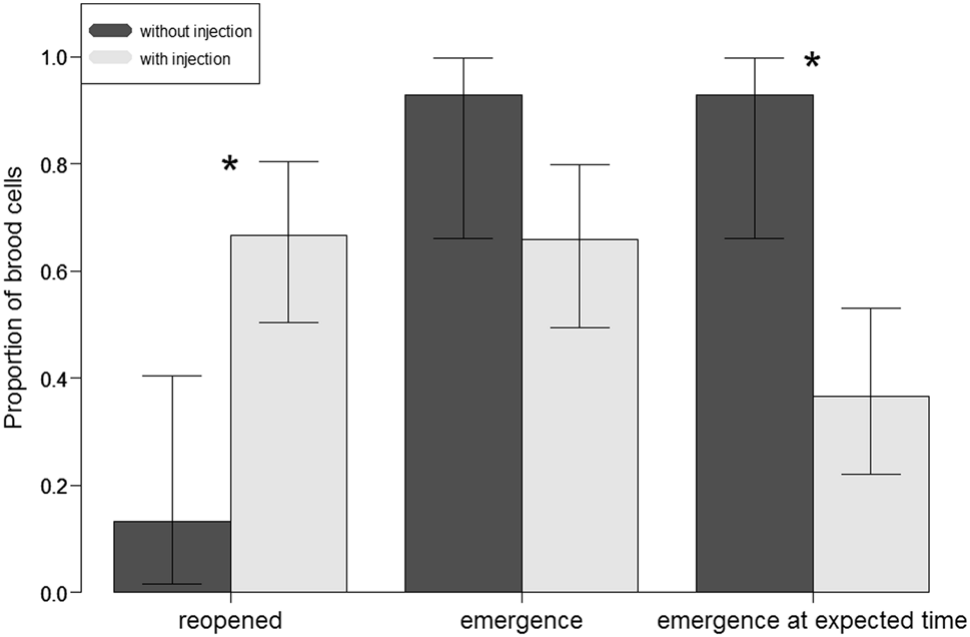
Injection of any of the treatments into a brood cell, including distilled water, caused differences in the probability of the foundress reopening the cells after closure, and of broods finishing development within the expected time frame. Foundresses were more likely to reopen brood cells that had been injected (GLMM, *p* = 0.00193). Injection did not affect significantly the chances of having an adult emerge from the brood cell (GLMM, *p* = 0.0797). However, it did affect the chance of finishing development on time (GLMM, *p* = 0.00417). *Error bars* represent 95 % confidence intervals from a simple binomial test. They are plotted to represent the variation of the data, but their significance is not directly related to the statistical model used for the analysis

Within the first week of our failed nourishment experiments, there were unusual patterns of adults reopening injected brood cells. Figure 2 shows that the injected brood cells were significantly more likely to be reopened by the foundresses than cells that had not been injected (GLMM, *p* < 0.01), indicating a change in the behavior of foundresses toward brood cells following injections.

**Fig. 3:**
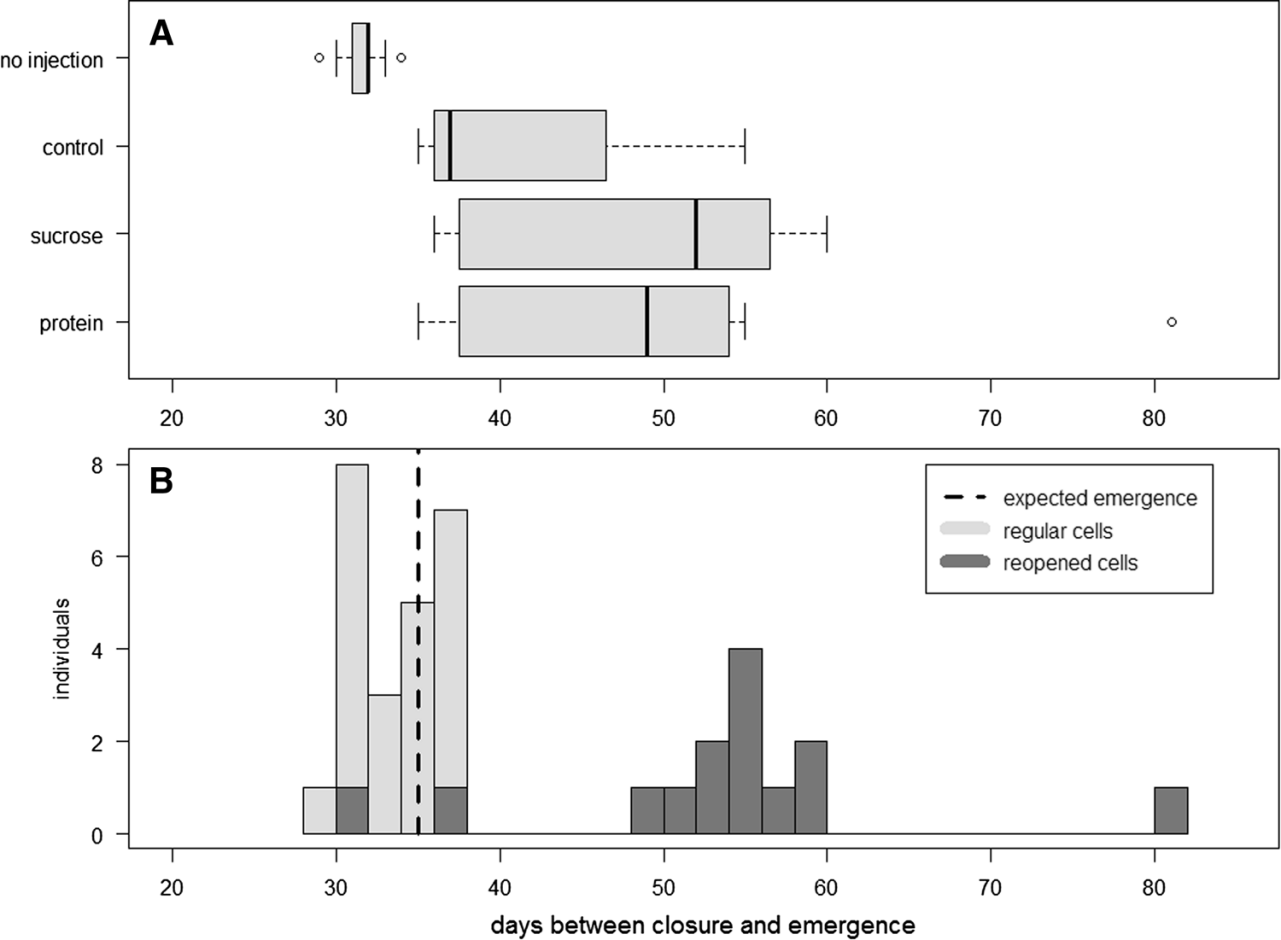
Injection treatments triggered a second oviposition event in some of the brood cells. **a** Injected brood cells had a significantly longer time between closure of the brood cell and emergence of the adult (GLM, *p* = 0.0236). **b** Histograms of the data for brood cells that were not reopened (normal cells) and reopened brood cells. Data for normal cells cluster around the expected time of development, while those of the reopened cells (*dark gray bars*) cluster many days after the expected time of development. For the reopened cells, if we consider only the time between the second closure and emergence, they also cluster around the expected time of development. The outlier in protein treatment (**a**) can be seen as an isolated bar at the higher end of the histogram (**b**), which corresponds to a brood cell that was reopened twice even though it had been injected only once

Cells that were reopened contained larvae that had either died during or soon after the injection procedure or been aborted by the foundresses. Some cells were completely destroyed by the foundress: All contents (provisions, eggs or larvae) were removed. Other cells (68 % of the reopened ones) were reclosed with contents left in place. These resealed cells subsequently produced adult bees. Statistical analyses showed that neither of the experimental manipulations had a significant effect on the probability of adult emergence (Fig. 2). Comparisons between the development times of the larvae in manipulated or un-manipulated cells showed much faster development in the latter (Fig. 3a). The difference in development time is demonstrated by the two different clusters of data (Fig. 3b). First, there is a cluster around the expected time of development, and most of these cells had not been reopened. The second cluster was composed only of cells that had been reopened, and these were more likely to have been subjected to a treatment involving injection as mentioned before (Fig. 2). This was confirmed by the significant effect of reopening on development time (GLMM, *p* < 0.001). If we used the second brood cell closure (after reopening) as the starting point of development, this cluster of points overlapped with development time of cells that had not been reopened (Figure S1). Furthermore, when the analysis was restricted to cells that had finished development on time (<40 days), we found a significant effect of injection on the probability of emergence (*p* < 0.01; Fig. 2). This result suggests that females oviposited a second time in the same brood cells.

Following cell reopening, foundresses were more likely to remove pollen from the nest than other cell contents. Using changes in state of the brood cells, we characterized the nests in terms of the abundance of different brood cell types (i.e., actively being provisioned, closed, reopened or empty following emergence). The only state change that was a good predictor of finding pollen in the receptacle was the reopened state, whether analyzed as a binary variable (presence or absence, *p* = 0.00351) or as counts of brood cells (*p* = 0.0076). After deciding that the provisioned cell would not produce viable offspring, the foundresses evacuated the initial pollen provisions, provisioned these brood cells a second time, and then laid second eggs.

Foundresses also removed the feces of recently emerged larvae. The presence of successful brood cells (=recently emerged adult) was a good predictor of the presence of feces in the receptacle (*p* < 0.001). This means that foundresses express hygienic behavior and clean the nest cells by removing feces of the newly eclosed adults from the nest, which is confirmed by footage taken from our experimental nests (see online resource 1).

## Discussion

Provisioning strategy (i.e., mass *versus* progressive) has been used as the primary proxy for extended brood care in social insects, on the assumption that a physical barrier (a closed cell) precludes caregiving. Halictine bees are exemplary mass provisioners in that females of many species collect all the pollen and nectar that developing young need before laying eggs and seal off brood cells afterward. However, our results show that *M. genalis* foundresses actively respond to changes relating to offspring viability that occur inside their brood cells. This response implies that females have the capacity to perceive changes inside the brood cell, despite the fact that sealing up the brood cell formed a physical barrier between parents and offspring. This type of care is not evident from the usual developmental path of a *M. genalis* nest, because it was triggered by experimental events relating to brood development.

A solitary halictid bee (*Nomia melanderi*) likewise detects cells that have been infected with various fungal species, opens the cells and packs them with soil (Batra and Bohart 1969). Contrary to earlier assumptions, evidence has been accumulating on the occurrence of extended brood care in mass-provisioning halictine bees (reviewed in Plateaux-Quénu 2008). Plateaux-Quénu showed that reopening of brood cells occurs in 11 social halictines, and she added evidence for six more species. The behavior of only a small number of halictines has been studied in detail, so it is presently not possible to estimate the true frequency of this behavior nor its phyletic distribution. For example, Danforth and Eickwort (1997: p. 277) stated that of the roughly 470 species of the then-described species of Augochlorini (Halictidae), only 2.8 % of them are “studied in sufficient depth to provide a good picture of their social biology”. Even fewer species have been studied with methods appropriate to detect cryptic brood care. Our results provide experimental evidence for the occurrence of this behavior in 13 % of experimentally unmanipulated brood cells and 62 % of injected cells, but the frequency of cell reopening in natural nests of *M. genalis* is unknown. Moreover, the behavior of *Lasioglossum* (*Evylaeus*) *calceatum* inside the brood cells (Plateaux-Quénu 2008) might explain the patterns of emergence in our experiments. Females of *L. calceatum* remove the egg, larvae, and/or pollen ball when the brood is dead or diseased. In our study, we cannot be certain in specific cases whether the removed brood were dead, diseased or healthy, so the cues that triggered foundresses’ behavior are unknown. We do know, however, that females can recognize and remove corpses. At the end of our experiment, we killed a few of the last instar larvae, and this triggered females to reopen cells and remove dead larvae immediately (see online resource 2). The response was different from the one triggered by the manipulations, where reopening took on average 4 days after the manipulation was performed. Finding the cues that females use to assess the condition of their brood will deepen our understanding of the evolution of parental care.

Undertaking and hygienic behaviors are associated with complex social life. Undertaking behavior shows wide variation in social insects with large colonies and encompasses different mechanisms (Sun and Zhou 2013). This diversity is likely caused by selection pressures imposed by social life (Hamilton 1987; Cremer et al. 2007). Our results support the claims of Plateaux-Quénu (2008) that the hygienic behavior of removing feces is associated with increased social complexity, as foundresses did this as soon as adults had emerged (see online resource 1). Although detailed studies of solitary halictines are scarce, field observations indicate that on occasion cells are reused in some solitary nests (e.g., *Lasioglossum figueresi;* Wcislo et al. 1993). In other solitary and social halictid bees, contaminated cells are packed with soil but not reused (Batra and Bohart 1969; WTW pers. obs.).

Extended brood care has been proposed as a preadaptation for eusocial life (Plateaux-Quénu 2008). There are at least two proposed mechanisms by which parental care could lead to eusociality. Our results point to hygienic behavior as an important component of parental care in *M. genalis*. Foundresses recognize non-viable brood cells, and they remove feces from cells that produced adults. Social life brings with it new challenges in the struggle against pathogens, because close contact with conspecifics increases pathogen transmission rates (Hamilton 1987). Thus, species that exhibit hygienic behavior as a form of extended brood care might be able to reduce the costs associated with social life while reaping the benefits of associated increases in productivity. There are alternative ways to deal with increased pathogen pressure. Parental care could have a more direct causal role in the benefits of social life, as found in assured fitness returns models (AFR) (Gadagkar 1990; Queller 1994). AFR proposes that social life is beneficial, because if a nest foundress dies, helpers in the colony would provide the necessary care for the offspring to complete their development (Gadagkar 1990; Queller 1994). The prerequisite of receiving parental care to finish development is one driver of sociality. AFR have been found in *M. genalis* in the form of reduced predation pressure on the brood under eusocial conditions (Smith et al. 2003, 2007).

Finally, we reiterate that our experiments were not originally designed to study cryptic extended brood care of *M. genalis*. They were designed to assess the effect of nutrition on social behavior (cf. Hunt and Nalepa 1994; Kapheim et al. 2011). Thus, the choice of treatments was not designed to shed light on the specific triggers for extended brood care. This behavior is latent in *M. genalis* and can become manifest under the right conditions, but additional studies are now needed to determine the specific conditions and triggers for its expression. Many behaviors might have evolved as a reaction to conditions that are not often encountered, but may have serious consequences for the organism. Therefore, such cryptic behaviors may be difficult to spot but may, nevertheless, play an important role in the evolution of sociality. So, we should not confuse the absence of evidence with evidence of absence.

## Electronic supplementary material

Effect of experimental treatments on the probabilities that i) the brood cells were reopened by the foundress, ii) that adults eventually emerged, and iii) that adults emerged within the reported developmental time of *M. genalis* (< 40 days). Treatment had a significant effect on the probability of reopening and the probability of emergence on the expected time, but not on the probability of emergence. *Post-hoc* comparisons showed that the protein treatment is significantly different to the ‘no injection’ treatment at the 95 % confidence both in the probability of reopening and emergence on expected time (*p* = 0.0397, *p* = 0.0211 respectively). All the other treatments were not significantly different from each other in any of the three measures. B). Histogram of the number of days between closure and emergence for all the brood cells. Dark grey bars correspond to the same measure but counted between the second closure and emergence (for cells that were reopened and reclosed). They overlap with the cells that were not reopened (DOCX 24 kb)

Footage of a female performing hygienic behavior, which is removing the feces of a newly emerged bee. Two females move around the brood cell. First, the foundress goes into the brood cell, and places the feces in the main tunnel. Later, a second female (a worker), moves the feces out of the scope of the camera. The waste was later removed completely from the nest (MPG 11,560 kb)

Footage of a female performing undertaking behavior, which is removing a corpse from the nest. A last instar larva was killed with the needle of a syringe. Within 30 minutes after the injection the foundress reopened the brood cell containing the dead larva. In the video she takes the dead larva out of the brood cell to the main tunnel, and then moves it towards the main entrance; later it was removed from the nest (MPG 56,912 kb)
