# Supplementary figures and images for: Cryptic extended brood care in the facultatively eusocial sweat bee Megalopta genalis
Source: Insectes Soc. 2015 Apr 26;62(3):307–13. doi: 10.1007/s00040-015-0409-3 (PMC4469088; doi:10.1007/s00040-015-0409-3)

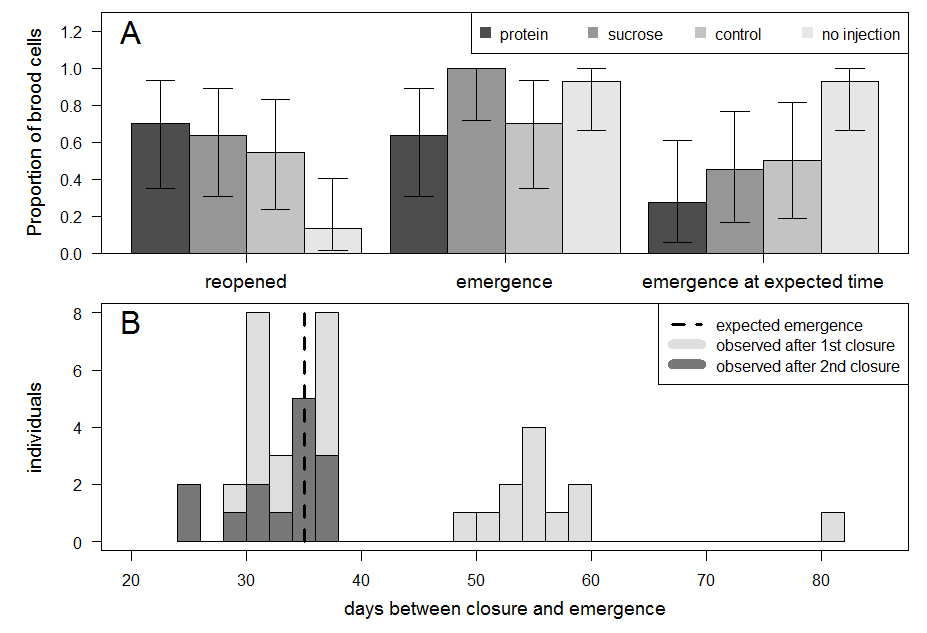

Supplement: Supplementary file 1 — Effect of experimental treatments on the probabilities that i) the brood cells were reopened by the foundress, ii) that adults eventually emerged, and iii) that adults emerged within the reported developmental time of M. genalis (< 40 days). Treatment had a significant effect on the probability of reopening and the probability of emergence on the expected time, but not on the probability of emergence. Post-hoc comparisons showed that the protein treatment is significantly different to the ‘no injection’ treatment at the 95 % confidence both in the probability of reopening and emergence on expected time (p = 0.0397, p = 0.0211 respectively). All the other treatments were not significantly different from each other in any of the three measures. B). Histogram of the number of days between closure and emergence for all the brood cells. Dark grey bars correspond to the same measure but counted between the second closure and emergence (for cells that were reopened and reclosed). They overlap with the cells that were not reopened (DOCX 24 kb) [file 40_2015_409_MOESM1_ESM.docx]
